# Exploring the utilization and deficiencies of Generative Artificial Intelligence in students’ cognitive and emotional needs: a systematic mini-review

**DOI:** 10.3389/frai.2024.1493566

**Published:** 2024-11-11

**Authors:** Elvis Ortega-Ochoa, Josep-Maria Sabaté, Marta Arguedas, Jordi Conesa, Thanasis Daradoumis, Santi Caballé

**Affiliations:** ^1^Doctoral School, Universitat Oberta de Catalunya, Barcelona, Spain; ^2^Computer Science, Multimedia, and Telecommunication Faculty, Universitat Oberta de Catalunya, Barcelona, Spain; ^3^Department of Cultural Technology and Communication, University of the Aegean, Μytilene, Greece

**Keywords:** cognition, emotions, Generative Artificial Intelligence, Large Language Models, systematic mini-review

## Abstract

Despite advances in educational technology, the specific ways in which Generative Artificial Intelligence (GAI) and Large Language Models cater to learners’ nuanced cognitive and emotional needs are not fully understood. This mini-review methodically describes GAI’s practical implementations and limitations in meeting these needs. It included journal and conference papers from 2019 to 2024, focusing on empirical studies that employ GAI tools in educational contexts while addressing their practical utility and ethical considerations. The selection criteria excluded non-English studies, non-empirical research, and works published before 2019. From the dataset obtained from Scopus and Web of Science as of June 18, 2024, four significant studies were reviewed. These studies involved tools like ChatGPT and emphasized their effectiveness in boosting student engagement and emotional regulation through interactive learning environments with instant feedback. Nonetheless, the review reveals substantial deficiencies in GAI’s capacity to promote critical thinking and maintain response accuracy, potentially leading to learner confusion. Moreover, the ability of these tools to tailor learning experiences and offer emotional support remains limited, often not satisfying individual learner requirements. The findings from the included studies suggest limited generalizability beyond specific GAI versions, with studies being cross-sectional and involving small participant pools. Practical implications underscore the need to develop teaching strategies leveraging GAI to enhance critical thinking. There is also a need to improve the accuracy of GAI tools’ responses. Lastly, deep analysis of intervention approval is needed in cases where GAI does not meet acceptable error margins to mitigate potential negative impacts on learning experiences.

## 1 Introduction

Artificial intelligence (AI), since its inception in the mid-20th century, has evolved from basic systems to advanced models like Generative AI (GAI) and Large Language Models (LLMs), capable of generating human-like responses and personalizing learning. Despite advancements in educational technology, a critical examination of how GAI and LLMs uniquely address learners’ nuanced cognitive and emotional demands remains unexplored. Existing literature, such as the studies by Yan et al. (2024) and Bahroun et al. (2023), has laid a significant foundation for understanding the application of LLMs and GAI within educational settings. These reviews have broadly covered the deployment and impact of these technologies, focusing on their technical implementation, overall effectiveness, and associated ethical and practical challenges, such as privacy and system transparency. However, these studies predominantly concentrate on general technological and ethical implications, leaving a gap in the specific exploration of GAI’s capacity to meet learners’ individual cognitive and emotional needs adaptively.

Given the identified gap, this systematic mini-review addresses the research question: How have GAI tools been utilized to cater to learners’ emotional and cognitive needs within educational settings, and what are the limitations in their adaptive response to these needs? This focus is pivotal as it can improve academic outcomes, considering the vital role of emotional well-being and cognitive engagement in successful learning experiences (Pekrun, 2017; Pekrun et al., 2017; Vygotsky, 1978). By shedding light on these limitations, the review aims to foster academic discussions, guide future technological advancements, and inform education stakeholders about the potential and constraints of current GAI applications in real-time adaptive learning environments.

The remaining sections are outlined as follows: the second section explains the methodology employed in this systematic mini-review; the third section presents the review’s results; and the fourth section interprets the results, addresses limitations, and presents implications. This mini-review considers inclusion criteria for studies mentioning ethical aspects in their records and reports. It is worth mentioning that the detailed information on the GAI tools and the ethical aspects of the studies included will be presented in future articles to provide a clear description of each component without affecting their comprehensibility, given the limited publication space in each report.

## 2 Method

This mini-review followed the Guidelines for performing Systematic Literature Reviews in Software Engineering (Kitchenham and Charters, 2007). A systematic review aims to collate evidence that meets pre-specified eligibility criteria to answer a specific research question while minimizing bias using explicit, systematic methods documented in advance with a protocol (Higgins et al., 2019).

### 2.1 Eligibility criteria

There are several inclusion criteria. Reports must be published as journal or conference papers. Studies that conducted empirical research on the deployment of GAI tools, had a practical application of these tools within the teaching and learning process, considered the learner’s emotional and cognitive needs in the design, development, or design of the experience, and exposed ethical considerations, responsive use practices or gaps in ensuring ethical deployment of GAI in education. In addition, reports must be published as journal or conference papers from 2019 to 2024 and be written in English.

Conversely, there are seven exclusion criteria. Reports published as reviews or book chapters (EC1). Studies that did not use GAI tools (EC2), did not have a practical application in education, either academic or auxiliary (EC3), did not reference the learner’s emotional and cognitive needs in the intervention (EC4), or did not expose ethical considerations, responsive use practices or gaps in ensuring ethical deployment (EC5). In addition, reports published before 2019 (EC6) or in languages other than English were excluded (EC7).

The information sources were Scopus and Web of Science; the last date the search was launched was June 18, 2024. Query: ((“educat*” OR (“learn*” AND NOT “data learning” AND NOT “machine learning” AND NOT “deep learning” AND NOT “federated learning”) OR “e-learning” OR “elearning” OR “teach*”) AND (“generative AI” OR “generative artificial intelligence” OR “artificial intelligence” OR “artificial neural network” OR “machine intelligence” OR “machine learning” OR “deep learn*”) AND (“emotion*” OR “affecti*” OR “empath*” OR “sentiment*” OR “feel*” OR “mood”) AND (“ethic*” OR “moral*” OR “*bias*” OR “right*” OR “priva*” OR “*equit*” OR “fair*”)).

### 2.2 Selection process

Firstly, duplicate records were identified and removed before screening. Secondly, two screeners conducted a preliminary screening process independently, focusing on the title, abstract, and keywords to assess adherence to the eligibility criteria. The first exclusion criterion was recorded if the record did not meet the criteria. In instances of discrepancy, a consensus was reached through discussion, and inter-rater reliability was quantified using Cohen’s kappa coefficient. Finally, one screener read the complete reports comprehensively to ensure compliance with the eligibility criteria.

### 2.3 Data collection process

The selected reports underwent a two-phase reading process to gather information. This information was systematically cataloged in an Excel spreadsheet (XLS format). The data encompasses the tools (name and description), application context in the educational system, tool objectives and tasks, the specific emotional and cognitive needs of students addressed, ethical considerations, practices of responsible use, any identified gaps to adaptively respond to the learner’s emotional and cognitive needs and ensure ethical deployment of GAI in education. The data items presented in this report are emotional and cognitive needs. Cognitive needs refer to the mental processes required for learning, such as memory, attention, problem-solving, and comprehension. Emotional needs involve the requirement for support, understanding, and a safe, nurturing environment that fosters their emotional well-being and resilience. With the data already collected, a comparative analysis was delineated to synthesize the results.

## 3 Results

**Figure 1:**
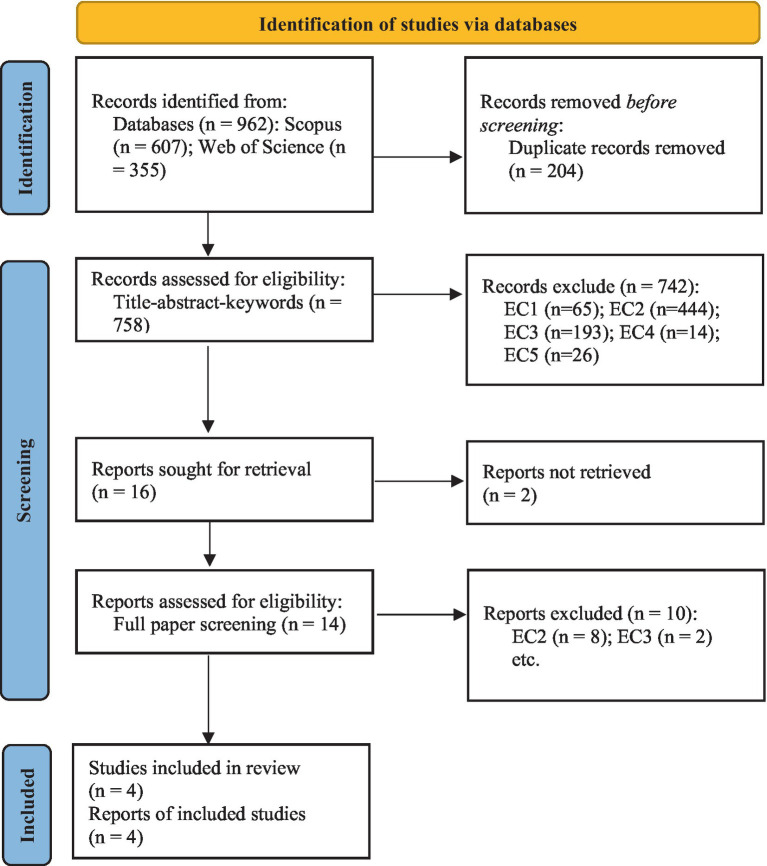
PRISMA 2020 flow diagram for new systematic review, which included searches of databases. EC: see inclusion and exclusion criteria section for an explanation of each eligibility criteria. Adapted from Page et al. (2021).

The initial search yielded a total of 962 records. After removing duplicates, 758 records remained for screening. Records were first screened by title, abstract, and keyword based on the inclusion criteria, which led to the exclusion of 742 records. The pairwise agreements between screeners exhibited almost perfect reliability, achieving a Cohen’s Kappa score of 0.96 for all the records. This score indicates an exceptional level of concordance between the screeners. Sixteen reports were sought for retrieval; however, two of them were not retrieved. The remaining 14 full-text articles were assessed for eligibility, resulting in 10 exclusions. Several reports that initially appeared to meet the inclusion criteria were excluded upon full-text review. For example, Jo (2024) mentioned a GAI tool; however, this tool is not part of the intervention process because this study is only a life-experience survey of a non-experimental design. After careful review, four reports containing four studies were included in this systematic mini-review. Figure 1 illustrates the flow of information through the different phases of the search and selection process, from the number of records identified to the studies included in the review.

### 3.1 Study characteristics

The studies included are Aure and Cuenca (2024; S1), Qureshi (2023; S2), Valový and Buchalcevova (2023; S3), and Walan (2024; S4). The studies use several GAI tools, mainly ChatGPT, to enhance learning. S1 and S4 are journal papers, while the remaining are conference papers. Table 1 shows the results of individual studies, precisely the cognitive and emotional needs addressed and their gaps.

**Table 1:** Cognitive and emotional needs addressed and their gaps.

ID	GAI tool name	Cognitive and emotional needs addressed	Gaps during response to the cognitive and emotional needs
S1	1. ChatGPT 2. Perplexity 3. Claude AI 4. Buzz Captions 5. Elicit	1. Emotional regulation and awareness: the study highlights the role of generative AI in supporting students’ social–emotional learning by helping them manage stress and maintain emotional well-being during the research process. AI tools offered personalized assistance that mitigated overwhelming feelings and helped students focus and structure their thinking, reducing anxiety and enhancing their emotional resilience. 2. Cognitive support and development: AI tools were utilized to enhance students’ cognitive engagement by providing brainstorming assistance, improving comprehension through simplifying complex texts, and aiding in organizing and articulating research findings. This boosted students’ research efficiency and deepened their understanding and critical thinking skills.	1. Critical thinking and independent analysis: despite the cognitive benefits, there remains a gap in ensuring that AI tools do not replace students’ critical thinking and independent analytical skills. The study notes instances where students might overly rely on AI for tasks requiring deeper cognitive engagement, potentially undermining their critical analytical skills development. 2. Emotional dependency: on the emotional front, the study acknowledges the risk of students developing a dependency on AI tools, which could affect their self-efficacy and ability to tackle challenges independently.
S2	ChatGPT	1. Enhanced engagement: ChatGPT in the curriculum aims to enhance student involvement and engagement by providing interactive and immediate feedback and solutions to programming problems. 2. Support for independent learning: ChatGPT is an on-demand resource that students can use to overcome challenges in understanding complex programming concepts, thereby supporting their cognitive development independently of direct instructor intervention. 3. Assistance with problem solving: the AI tool assists students by generating code snippets and step-by-step guides for solving programming tasks, which helps them grasp complex algorithms and coding techniques.	1. Understanding and accuracy: the document highlights a significant gap in ChatGPT’s ability to comprehend problems correctly and generate accurate code solutions, particularly as the complexity of problems increases. Students face issues with code that does not compile or fails to meet the problem requirements. 2. Depth of knowledge: ChatGPT, while helpful, cannot deeply understand the underlying concepts it discusses or the code it generates. This can lead to superficial learning where students might not fully grasp the core principles of computer science they are studying. 3. Dependency and misuse: there is a gap in ensuring that students use AI tools like ChatGPT appropriately without becoming overly dependent on them for solutions, which could hinder their learning process and problem-solving skills.
S3	1. GitHub Copilot 2. ChatGPT	1. Emotional safety and anxiety reduction: the study highlights that AI-assisted programming tools can significantly reduce anxiety and stress in students by providing immediate feedback and reducing the fear of failure. This supportive environment helps to bolster students’ confidence as they learn to program. 2. Engagement and motivation: AI tools are shown to enhance engagement through interactive and personalized learning experiences. This addresses students’ need for stimulation and helps maintain their interest and motivation in learning programming. 3. Cognitive load management: by automating routine aspects of coding, AI tools help manage students’ cognitive load, allowing them to focus on more complex problem-solving and creative aspects of programming. This addresses their need for cognitive balance, preventing overload and burnout.	1. Personalization gaps: the document discusses that while AI tools offer some level of personalization, there is a significant gap in adapting to individual learning paces and styles. The tools cannot fully understand and adapt to individual emotional responses, hindering personalized learning experiences. 2. Emotional connectivity: the study identifies a gap in the emotional connection between students and AI tools. Unlike human mentors, AI tools cannot provide empathetic support or understand nuanced emotional cues, which are crucial for emotional and psychological well-being. 3. Depth of cognitive support: while AI reduces cognitive load, there is a gap in supporting deeper cognitive processes like critical thinking and problem-solving in unstructured tasks. AI tools often focus on syntax and basic errors but less on logic or algorithmic creativity, which is essential for advanced programming skills.
S4	1. ChatGPT 2. DALL·E	1. Emotional support: students found AI helpful in supporting their studies and practical tasks, like aiding in exam preparation or potentially assisting with chores if they were unable to perform them due to illness. They expressed a mix of positive sentiments towards the capabilities of AI. 2. Cognitive engagement: students interacted with AI as an educational tool, exploring and learning about various subjects. AI’s role in enhancing their understanding and providing new ways to engage with content was highlighted.	1. Adaptive emotional response: one significant gap is AI’s inability to adapt responses based on emotional cues. Students noted AI’s lack of human-like emotional responses, which could be crucial in making interactions more personalized and supportive. 2. Cognitive development: AI cannot foster more profound cognitive skills comprehensively. While AI aids learning and task completion, its role in developing critical thinking or problem-solving skills independently was less evident.

### 3.2 Results of syntheses

#### 3.2.1 Cognitive and emotional needs addressed

This synthesis examines how GAI tools cater to cognitive and emotional needs in educational contexts, mainly focusing on enhancing engagement and emotional regulation. Regarding cognitive needs addressed, Qureshi (2023) demonstrated the integration of ChatGPT in curriculum delivery, notably increasing student engagement through interactive and responsive learning environments. The instant feedback GAI tools provide fosters a more captivating educational experience, heightening student interest and participation. As for emotional needs addressed, Aure and Cuenca (2024) explore how GAI tools aid emotional regulation during learning sessions. Identifying students’ emotional states and tailoring content accordingly, these tools help maintain a focused and positive learning atmosphere. Additionally, GAI tools are crucial in providing emotional safety and support, which is essential for a conducive learning environment. Valový and Buchalcevova (2023) highlighted using GAI to reduce anxiety and promote emotional safety, especially during assessments. By offering a non-judgmental and supportive interface, GAI tools encourage students to express their concerns freely, facilitating a safe space for learning without fear of negative consequences. Moreover, Walan (2024) notes that GAI tools offer emotional support by recognizing and responding to signs of distress or disengagement among students.

#### 3.2.2 Gaps during response to the cognitive and emotional needs

This synthesis of identified gaps in GAI across four studies emphasizes the need to enhance GAI’s capability to foster critical thinking and encourage independent analysis. Aure and Cuenca (2024) underscore a significant gap in how GAI supports learners in developing critical thinking skills, pointing out that learners often depend excessively on GAI outputs. This overreliance may impede their ability to analyze and form conclusions independently. Another core issue highlighted in the studies is the GAI’s limited understanding and accuracy, which affects the quality of interactions between the AI and students. Qureshi (2023) identifies problems with GAI’s ability to accurately interpret and respond to the depth of students’ questions and emotional cues. These shortcomings can lead to responses that are either irrelevant or incorrect, disrupting the learning process and potentially causing confusion among learners. Furthermore, Valový and Buchalcevova (2023) and Walan (2024) highlighted critical gaps in GAI’s personalization and emotional intelligence. Valový and Buchalcevova (2023) discuss how, despite being designed to adapt to individual learning profiles, GAI tools fail to deliver a genuinely personalized learning experience, especially in recognizing and adapting to each learner’s unique emotional and cognitive states. Similarly, Walan (2024) observes that GAI tools are inadequate in responding to learners’ emotional states, which could undermine the emotional support vital for effective learning.

## 4 Discussion

This mini-review investigated how GAI tools meet cognitive and emotional needs. GAI tools have shown considerable promise in enhancing student engagement and emotional regulation. For instance, ChatGPT improves engagement through interactive learning environments that offer instant feedback, thus maintaining high student interest and participation (Qureshi, 2023). Similarly, GAI tools effectively aid emotional regulation by identifying and responding to students’ emotional states, fostering a positive learning atmosphere (Aure and Cuenca, 2024). These findings are consistent with previous reviews, which report that integrating GAI offers substantial opportunities for enhancing educational practices and improving learning outcomes (Bahroun et al., 2023). While some of these students’ needs resonate with those proposed in prior positioning works (e.g., teaching support, feedback, content generation, and recommendation) (Yan et al., 2024), novel directions such as automatic emotion regulation further indicated the potential of GAI tools. Despite these advancements, several gaps remain in the capabilities of GAI tools to adaptively respond to learners’ needs. A critical area of concern is the development of critical thinking skills, for example, an overreliance on GAI outputs, potentially hindering learners’ abilities to analyze independently (Aure and Cuenca, 2024). Additionally, there are issues with the accuracy of GAI’s responses, noting occasional misinterpretations that can disrupt learning and confuse students (Qureshi, 2023). Furthermore, there are limitations in GAI’s personalization and emotional intelligence; these tools often fail to deliver genuinely personalized experiences and are sometimes inadequate in providing the necessary emotional support (Valový and Buchalcevova, 2023; Walan, 2024). These findings are consistent with previous reviews, which report a low level of technology readiness, where the innovations have yet to be fully integrated and validated in authentic educational contexts (Yan et al., 2024).

### 4.1 Limitations and implications

This mini-review presents several limitations and implications. From the perspective of the included studies, their results may not be generalizable beyond specific versions of GAI, for example, ChatGPT (versions 3.5 and 4) and GitHub Copilot (from July 2021 to June 2023). Furthermore, these studies are cross-sectional and involve a small number of participants. The review’s stringent eligibility criteria, which require explicit mention of terms related to GAI, education, and emotions in the records, along with ethical considerations and responsible use, may have excluded relevant studies that do not discuss these topics in their record but do address them in the full report. Additionally, implications for practice include the need to consider teaching and learning strategies that utilize GAI while simultaneously promoting critical thinking skills among students. Moreover, enhancing the accuracy of GAI tool responses is essential, and all stakeholders must collaborate on this front. The actual capability of GAI to provide personalized cognitive and affective support should be thoroughly reported. Approval of interventions should be deeply analyzed in cases where this does not meet acceptable error thresholds to prevent adverse effects on the learning experience. Future research should clearly define pedagogical strategies to foster critical thinking when using GAI. Key avenues for enhancing GAI tool accuracy and minimizing errors include improving data quality and diversity, advancing model architectures, fostering robustness and generalization, employing cross-validation with external sources, integrating human oversight, enhancing system explainability, conducting adversarial training, and quantifying uncertainty in AI predictions. Lastly, future research should assess the extent to which cognitive scaffolding and emotion regulation strategies are integrated into GAI tools.
